# GLYCOSAMINOGLYCANS AND PROTEOGLYCANS IN PALMAR FASCIA OF PATIENTS WITH DUPUYTREN

**DOI:** 10.1590/1413-785220162402154342

**Published:** 2016

**Authors:** Priscilla Carneiro Hirai Nascimento, Elsa Yoko Kobayashi, Luiz Guilherme de Saboya LENZI, João Baptista Gomes dos Santos, Helena Bonciani Nader, Flávio Faloppa

**Affiliations:** 1. Universidade Federal de São Paulo (Unifesp), Escola Paulista de Medicina, Department of Orthopedics and Traumatology, São Paulo, SP, Brazil

**Keywords:** Dupuytren contracture, Glycosaminoglycans, Proteoglycans, Fascia

## Abstract

**Objective:**

: To evaluate and compare the behavior of glycosaminoglycans (GAGs) in Dupuytren disease (DD).

**Methods:**

: This is an experimental study with 23 patients diagnosed with DD. Tissue collected through fasciectomy with incision type Brunner or McCash were evaluated by electrophoresis for identification of GAGs. The quantification was carried out by immunofluorescence and dosage of proteins for different types of glycosaminoglycans. The results were expressed in percentage and statistically evaluated.

**Results:**

: A significant increase was observed through eletrophoresis in GAGs, as compared to the control (p<0.05). Immunofluorescence of hyaluronic acid was reduced (23 times) when compared to the control (p<0.0001).

**Conclusion:**

: An increase of sulfated GAGs in Dupuytren's disease, mainly dermatan sulfate, was evident from our results, as well as a pronounced decrease of hyaluronic acid in the palmar aponeurosis from the same patients. Level of Evidence III, Case-Control Study.

## INTRODUCTION

Dupuytren's disease (DD) is a fibroproliferative disorder that can cause progressive and permanent flexion contracture of the fingers.^1^ It is a debilitating condition characterized by fibrotic strands with consequent thickening of the palmar fascia, due to excessive collagen thick fibers (I and III).^1^^,^ 2

In Dupuytren's disease, the main cells involved are myofibroblasts. These cells have both fibroblasts and smooth muscle cell characteristics, possessing the ability to contract actively. Contractility of myofibroblasts may be affected by prostaglandins, which are present in the nodes and, thus,influence the disease, as well as other growth factors and proteins involved in fibrotic processes do, such as glycosaminoglycans (GAGs), which are increased in pathological processes with such characteristics. This abnormal cellular behavior can lead to changes in the extracellular matrix (ECM) and, consequently, changes in the conjunctive tissue array.^3^^,^ 4

The extracellular space is often filled by so-called fibroconnective extracellular matrix components. It consists, in varying proportions, of collagen, elastic fibers, proteoglycans (PGs), glycosaminoglycans (GAGs) and cellular elements, which are organized forming a network, which is partly responsible for the great morphological and functional diversity of the various tissues.^5^^-^ 7 GAGs were initially called mucopolysaccharides. They differ from each other according to the type of hexamine and nitrogen free sugar, according to the degree of sulfation and the position in which they are sulfated. This variety results in the following GAGs: heparin, heparan sulfate (HS); chondroitin sulfate (CS); dermatan sulfate (DS); keratan sulfate (KS) and hyaluronic acid (HA).^3^^,^ 4 The GAGs, except for the HA, are synthesized as proteoglycans.^5^

Despite the advances in knowledge and research on the metabolism of GAGs, little is known about the changes occurring in Dupuytren's disease and Carpal Tunnel Syndrome, as well as their levels under pathological conditions.^3^^,^ 6 ^,^ 7 The aim of this study was to evaluate and compare the behavior of GAGs in Dupuytren's disease.

## MATERIALS AND METHODS

This is an experimental study, conducted at the Discipline of Hand and Upper Limb Surgery at the Department of Orthopedics and Traumatology, EPM,UNIFESP, from July 2005 to September 2006. The present study included 23 patients diagnosed with DD, who signed a Free and Informed Consent Term. As control group, samples of normal palmar aponeurosis were taken from patients undergoing carpal tunnel release. The project was approved by the Ethics Committee (N° 48734315.2.0000.5505).

The inclusion criterion was patients with surgical indication for DD.Individuals who had recurrence of the disease were excluded from the study.

Samples were collected through fasciectomy with Brunner type incisions (multiple "V") or by McCash technique, also known as open palm technique,where healing occurs by secondary intention.

### Extraction and identification of sulfated glycosaminoglycans (GAGs) 

The tissues were washed in phosphate buffered saline (PBS) to remove excess glutaraldehyde and chopped in acetone. The obtained fragments were dehydrated and kept for 24h at 4°C. The ketone powder obtained was weighed individually. The extraction was performed for each tissue separately. The dry powder was subjected to proteolysis with 4 mg/ml maxatase in 50mM Tris-HCl buffer at 60°C, pH 8.0. A NaCl solution final concentration 1M was added to the remaining volume. Nucleic acids peptides were precipitated with 90% trichloroacetic acid at final concentration 10% in an ice bath. After precipitation, the material was centrifuged for 20 min and the supernatant collected. To the supernatant two volumes of methanol were added to precipitate the GAGs. Precipitation was performed at -20°C (18-24h). The material was centrifuged and the precipitate containing the GAGs ​​was rehydrated in distilled water at a ratio of 5 mg of acetone powder to 20 µL distilled water.^8^

The compounds were applied to 0.2 cm thick agarose gel sheets (0.6%) in0.05M 1,3-diaminopropane acetate buffer pH 9.0. The electrophoresis was performed in a refrigerated box at 4°C and 100 mV for about one hour or until the proper migration occurred, indicated by red cresol dye.

After the electrophoresis, the glycosaminoglycans were precipitated into the gel with 0.1% cetavlon (cetyltrimethylammonium bromide) for at least 2h. After drying under heat and ventilation, the gel was stained with 0.1% toluidine blue solution in 1% acetic acid and 50% ethanol.

Glycosaminoglycans were identified by comparing the electrophoretic migration of sample with known and purified standards. These same patterns were used for the quantitative determination of the compounds by optical densitometry at 525 nm.

This is a fluorometric method for determining hyaluronic acid in biological fluids, using binding probes to HA.^9^ The probe is isolated from bovine nasal cartilage and consists of globular agrecam (a proteoglycan formed by a protein backbone to which strands of keratan sulfate and chondroitin sulfate are attached) and HA-binding protein. The probe is stabilized in ELISA plates, like a capture antibody such as a biotinylated probe, working, in the latter case, as a labeled secondary antibody.

To the ELISA plate with the adsorbed probe 100 µL/well of standard HA solutions of various concentrations (0 to 500 g/L) diluted in 0.05 M Tris-HCl assay buffer pH 7.75 and 1% bovine serum albumin (Amersham Life Science Ltd., Buckinghamshire, England) were added, besides sample solutions obtained from the aponeuroses diluted in the same test buffer, in triplicates.

The palmar aponeurosis were fixed in 10% formaldehyde in PBS and then included in Tissue Tek at minus 20°C. Then, they were cut in 8µm slices on a cryostat and placed on slides for immunofluorescence analysis.

The slides, after washing and digestion, were incubated with the primary antibody (1:100 byglican and decorin). Thereafter, the slides were kept at room temperature for 2h and washed with PBS.

The slides were, then, incubated with the secondary antibody (anti-decorin conjugated with AlexaFluor-488 and anti-byglican conjugated with Alexa fluor-594) for 2h at room temperature. After this step, the slides were washed five times with PBS and then incubated with the second primary antibody (ß-TGF) for 4h at room temperature and then washed and incubated with the second secondary antibody (anti-ß-TGF conjugated with Alexa-fluor-594) for 2h at room temperature.

Subsequently, the slides were washed with PBS, and nuclei identification was performed by incubation with 1: 1000 4,6-diamidino-2-phenylindole, dihidrocloride (DAPI, Molecular Probes, Eugene, OR, USA). Finally, slides were mounted in Fluoromont G (2:1 in PBS) and analyzed on a laser scanning confocal microscope.

## RESULTS

**Figure 1.**
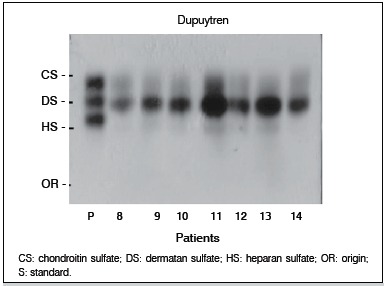
Electrophoretic behavior of GAGs extracted from aponeurosis of Dupuytren's disease patientsin PDA agarose buffer (patients 8 - 14).

**Figure 2.**
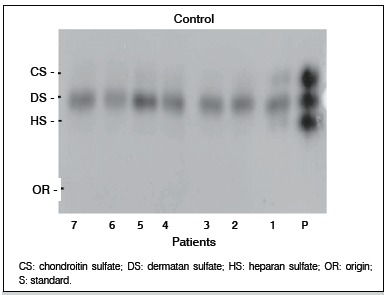
Electrophoretic behavior of GAGs extracted from aponeurosis of control patients in PDA agarose buffer (patients 1 - 7).

There was a greater expression of GAGs extracted from the aponeuroses compromised by Dupuytren's disease in the electrophoresis (Figure 1), when compared to the electrophoresis of control aponeuroses. (Figure 2)

Regarding the amount of total GAG (mg/g of dry weight), we found statistically significant differences values between Dupuytren and control samples (p <0.03), an increase of almost twice on Dupuytren samples.

Chondroitin sulfate (CS) was increased in Dupuytren samples, however, the difference was not statistically significant (p <0.06).

In contrast, the concentration of dermatan sulfate (DS) in the Dupuytren sample compared to the control group was significantly increase (p <0.03). However, heparan sulfate (HS) was the GAG ​​found in the smallest concentration, and there was no difference between samples.

The amount of hyaluronic acid (HA) was significantly lower in Dupuytren samples (23-fold) when compared to control samples (p <0.0001).

Regarding the percentage distribution, the GAG ​​found in the highest concentration in both samples was DS, but the difference between the two groups was not statistically significant (p <0.77). The second most prevalent GAG in the samples was CS, which also showed no statistically significant difference between samples (p <0.11). HS was found in small quantities in Dupuytren and control samples, and its percentage was similar in both groups regarding aponeuroses composition of (p <0.55).

As noted in Figure 2, aponeuroses from Dupuytren patients showed a marked increase of dermatan sulfate (DS) and a little more discreet chondroitin sulfate (CS) increase, as compared to the control. (Figure 1) Due to this result, we investigated the behavior of extracellular matrix proteoglycans of CS/DS (decorin and byglican), of these aponeuroses, in order to characterize these tissues' matrix. ß-TGF was also analyzed, as it binds to decorin.

**Figure 3.**
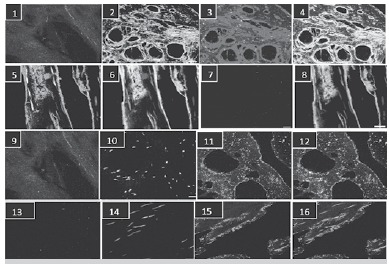
(1 - 4): Confocal microscopy; 1: negative control; 2: immunolabeling with anti-decorin polyclonal antibody; 3: immunolabeling with anti ß-TGF polyclonal antibody;4: overlap of images B and C; (5 - 8): immunolabeling of control patient; 5: negative control; 6: immunolabeling with anti-decorin polyclonal antibody; 7: immunolabeling for the ECM using anti ß-TGF polyclonal antibody; 8: overlap of images B and C, the nucleus was detected with DAPI; (9 - 12): immunolabeling of Dupuytren patient; 9: negative control; 10: immunolabeling with DAPI-detected nucleus; 11: immunolabeling with anti-byglican polyclonal antibody; 12: overlap of images B and C; (13 - 16): immunolabeling of control patient; 13: negative control; 14: identification of nucleus detected with DAPI; 15 immunolabeling with anti-byglican polyclonal antibody;16: overlap of images B and C.

In figure 3 we observe an increase in decorin labeling in tissue of Dupuytren patient (1 - 4) as compared to the control (5 - 8). The labeling of ß-TGF in both tissues is shown in Figure 3-3 and 3-7. Figures 3-4 and 3-8 show an overlap of decorin and ß-TGF, showing that anchoring of decorin in the extracellular matrix of the tissue occurs in the presence of ß-TGF. It is also possible to observe that these components have determined an edge marking for the analyzed tissue. The nucleus was visualized with DAPI (blue). In Figures 3, 9-12 and 13-16 we observe the immunostaining for byglican in control and Dupuytren tissues. It is possible to observe an increased expression of this proteoglycan in DD tissues (Figure 3, 9-12) as well as an edge arrangement. 

## DISCUSSION

There are few studies relating Dupuytren's disease with GAGs metabolism,^6^^,^ 7 but it is known that there are changes in the ECM due to this pathology, which contributes to conjunctive tissue arrangement.^5^

GAGs are important components of the extracellular matrix,locally secreted and organized into a network located on the cell surface. Until recently, it was believed that ECM of vertebrates served as an inert structure that stabilized the physical structure of tissues. It is now clear that the matrix plays much more active and complex role in regulating cell behavior, directly influencing differentiation, migration, adhesion, cell proliferation and modulation, among others.^10^

Dermatan sulfate is a GAG found in a wide variety of vertebrate tissues. It is widely distributed in the form of proteoglycans in many tissues such as cornea, sclera, skin, liver and spleen tissue.^11^ It was isolated from ECM of connective tissues which, together with collagen, elastin and other glycoproteins, plays an important role in maintaining the tissue's structural integrity.^12^

The literature described four-fold increase of DS of the palmar aponeurosis in DD patients.^12^ We found a marked increase of DS concentration in aponeuroses samples of Dupuytren patients. This was the most prevalent GAG in all samples, however, in those from DD patients, dermatansulfate was increased twice.

Besides chondroitin sulfate, DS may be the main GAG associated to collagen, which is involved with this protein's organization and fibrogenesis.^13^ Its function is possibly associated with the organization, deposition rate and maintenance of collagen fibers.^14^ DS occurs in high concentrations in fibrous tissues rich in type I collagen.

Although the decrease of HA in the palmar fascia of patients with Dupuytren has been described, our study showed a much more significant reduction than the literature findings (23 times lower). Perhaps this is due to some differences between the literature and our work such as, for example, different sensitivity of the method used to quantify HA and the use of cadaver samples in other works.

Medical literature has described the rise of up to 11-fold the amount of chondroitin sulfate on the palmar aponeurosis of patients with Dupuytren.^14^^,^ 15 However, despite the higher amount of CS found in patients with Dupuytren than in the control groupin our study, this difference was not significant, diverging from other studies showing its increase in neoplastic tissues and other diseases such as arthrosis and chondrosarcoma. This may be a consequence of different sample size and GAG extraction methodology.^16^^,^ 17

However, heparan sulfate, which is located in the plasma membrane and the ECM^18^ and regulates interactions between cells and their environment, was slight increase in the Dupuytren samples, as also observed by other authors.^7^^,^ 15 Several evidences suggest that HS plays an important role in cell recognition and adhesion, growth control and angiogenesis.^19^

Histologically, Dupuytren's disease, also known as palmar fibromatosis, presents a behavior common to some cancers, such as fibroblast proliferation,^20^ proliferative and invasive growth,besides relapse. Fibroblasts found in Dupuytren's disease show *in vitro* properties similar to tumors', such as chromosomal abnormalities and binding capacity to monoclonal antibodies.^21^

As shown in an earlier work,^19^ the percentage distribution of sulfated GAGs in both groups was similar, CS was found in the highest concentrationand HS and in lowest.

In samples of Dupuytren's disease patients, we found all GAGs increased, but mostly CS and DS. Dermatan sulfate was increased almost twice in these samples. We do not know whether the increased quantity of those GAGs is related to decreased degradation or increased synthesis. The reports on this aspect are inconclusive, which is an important point to be studied.^15^

As a hypothesis, we suggest that Dupuytren's disease has some tumor characteristics such as infiltrative and proliferative behavior, besides presenting relapse. The altered GAGsmetabolism, such as increased CS and decreased HA also reinforce the idea of a tumor behavior, as noted by Wilbrand et al.,^21^ whose study found that 24% of DD patients developed a malignant tumor even after surgical treatment. However, some aspects are still to be researched through the metabolism of GAGs associated with the tumor behavior of this disease. We believe it should be possible to find a serum marker based on change in GAGs metabolism.

## CONCLUSION

It was evident the increase of sulfated GAGs in Dupuytren's disease, mainly dermatan sulfate, and a marked decrease of hyaluronic acid in the patients' palmar aponeurosis.
